# Peru GDP real-time dataset (1994–2025): Tracking three decades of revisions

**DOI:** 10.1016/j.dib.2026.112874

**Published:** 2026-05-22

**Authors:** Jason Josue Cruz, Diego Winkelried, Javier Torres

**Affiliations:** School of Economics and Finance, Universidad del Pacífico, Av. Salaverry 2020, Jesús María, Lima 15072, Peru

**Keywords:** Real-time macroeconomic statistics, GDP revisions, Emerging markets, Nowcasting, Vintage data, Data curation, Central bank publications, Economic indicators

## Abstract

This data article introduces a comprehensive real-time dataset (RTD) of Peru’s Gross Domestic Product (GDP) growth rates from 1994 to 2025. The dataset was constructed by systematically collecting and processing three decades of the Peruvian Central Reserve Bank (BCRP) Weekly Reports and compiled into over 1000 structured data files organized across three processing tiers (raw, input, and output). For the 1994–2012 period, data were digitized from archival hardcover volumes using Optical Character Recognition (OCR) with rigorous manual verification. Post-2013 data were integrated via automated web-scraping and PDF extraction pipelines from digital publications. The resulting dataset includes monthly, quarterly, and annual growth rates for aggregate GDP and eight economic sectors, organized in two complementary formats: vintage (rows indexed by sector and publication month; columns correspond to target periods) and release (rows correspond to target periods; columns index the hth published estimate for that period, given each economic sector). The collection includes variants for base-year adjustments and identifies major benchmark revisions. All data are provided in CSV format with comprehensive documentation. An open-source Python pipeline enables full reproducibility and future updates. This RTD serves as an important resource for researching GDP revision patterns, real-time forecasting accuracy, nowcasting model evaluation, and cross-country comparisons of statistical practices in emerging economies.

Specifications Table

**Table:** 

Subject area	Social Sciences
Specific subject area	Real-time macroeconomic data, GDP revisions, emerging market economic statistics
Type of data	Table, Raw, Analyzed, Filtered, Processed
Data collection	CSV files
Data collection description	Data collected from BCRP Weekly Reports (1994–2025). Pre-2013: scanned PDFs processed with an 11-step Tesseract OCR pipeline (demonstrated on year 2001, achieving 70.5% completeness), extensive manual verification, and 70+ cleaning functions. Post-2013: Selenium web scraping, Tabula-py PDF extraction, and an automated cleaning pipeline. Monthly GDP tables were systematically extracted, standardized, and organized by release date. Python-based processing included quality validation checks. Complete OCR code and demonstration outputs are publicly available.
Data source location	Institution: Universidad del Pacífico (Lima, Perú). City/Country: Lima, Peru. Geographic coordinates: $-12.0897^{\circ }$S, $-77.0428^{\circ }$W. Data source: Banco Central de Reserva del Perú (BCRP). URL: https://www.bcrp.gob.pe/publicaciones/nota-semanal.html
Data accessibility	Repository name: Zenodo. Data identification number: https://doi.org/10.5281/zenodo.18099975. Related code repository: https://github.com/JasonCruz18/peru_gdp_revisions. Code DOI (frozen release v1.0.0): https://doi.org/10.5281/zenodo.18792757. Instructions: Dataset freely accessible via DOI, with CSV files and README documentation.
Related research article	None

## 1 Value of the Data

• This is the first real-time GDP dataset for Peru and one of the few available for emerging markets. By providing systematic vintage data for an emerging market economy, it extends the scope of real-time analysis beyond the advanced economy focus of existing datasets.
• The dataset enables evaluation of real-time forecasting and nowcasting methods, allowing researchers to assess forecast accuracy using the information available at the time predictions were made rather than revised data. This is essential for developing robust nowcasting models for emerging economies, where data quality and revision patterns may differ substantially from those in advanced economies.
• The dataset facilitates rigorous analysis and statistical quality assessments by enabling the investigation of revision properties, such as bias, efficiency, and rationality. Furthermore, the longitudinal nature of the data allows for the clear identification of benchmark revisions and structural breaks caused by base-year adjustments. This informs best practices for data producers and helps researchers better understand data generation processes.
• Through a complete open-source Python pipeline, the dataset can be exactly replicated and seamlessly extended as new data becomes available. Its modular architecture further allows straightforward adaptation to other countries or statistical agencies with minimal modifications.
• By providing multiple formats structured for different research applications, the dataset accommodates a wide range of empirical strategies, allowing researchers to select the most appropriate variant for their specific analytical needs. The vintage format facilitates real-time analysis; the release format enables revision econometrics; benchmark indicators isolate methodological changes; and base-year adjustments account for structural breaks.
• Comprehensive documentation and quality assurance are ensured through fully detailed metadata, cleaning procedures, and validation checks. Dual OCR and automated extraction methods provide consistent coverage across three decades despite changing data formats, resulting in more than 1000 vintages spanning eight economic sectors.

## 2 Background

Real-time datasets preserve the information actually available to decision-makers at each specific time point, making them indispensable for forecast evaluation [1], revision analysis [2], [3], [4], and the identification of informational inefficiencies. While real-time data infrastructures based on the vintages framework [5], [6] are well established for advanced economies, comparable resources remain scarce for emerging markets, particularly in Latin America. Scarce exceptions can be found in the GDP revision literature, where researchers have built their own vintage datasets by harvesting periodic publications not available in easily processed form, closely paralleling the approach employed here. For example, Kishor [7] compiled GDP vintages from the Reserve Bank of India’s weekly statistical supplements, while Ramos [8] collected data from hard copies of press releases issued by Brazil’s statistical agency (IBGE). These cases underscore how scarce, yet valuable, real-time datasets are for emerging economies, and motivate the construction of a systematic vintage repository for Peru.

Peru offers a compelling case study. The Central Reserve Bank of Peru (BCRP) has consistently published GDP growth measures in its Weekly Reports for over three decades, spanning multiple commodity cycles, macroeconomic regimes, and institutional reforms. Despite this continuity, these releases had never been consolidated into a systematic real-time dataset, limiting empirical work on revision behavior and nowcasting performance for the Peruvian economy.

This dataset was constructed to fill that gap, providing a complete, historically consistent real-time record of Peruvian GDP growth from 1994 to 2025. This repository supports the decomposition of revisions into news and noise components [2], and the back-testing of forecasting models [9]. The construction required specialized procedures: pre-2013 releases, available only as scanned documents, required optical character recognition; post-2013 vintages were extracted automatically from digital archives.

## 3 Data Description

The dataset comprises 1132 CSV files organized in a three-tier structure that reflects the complete data processing pipeline: raw data (420 files), intermediate input vintages (696 files), and final output datasets (16 files). This structure enables users to reproduce the entire data workflow or to directly access analysis-ready datasets. All files are hosted on Zenodo (https://doi.org/10.5281/zenodo.18099975) with accompanying README documentation.

### 3.1 Dataset overview and file structure

**Fig. 1:**
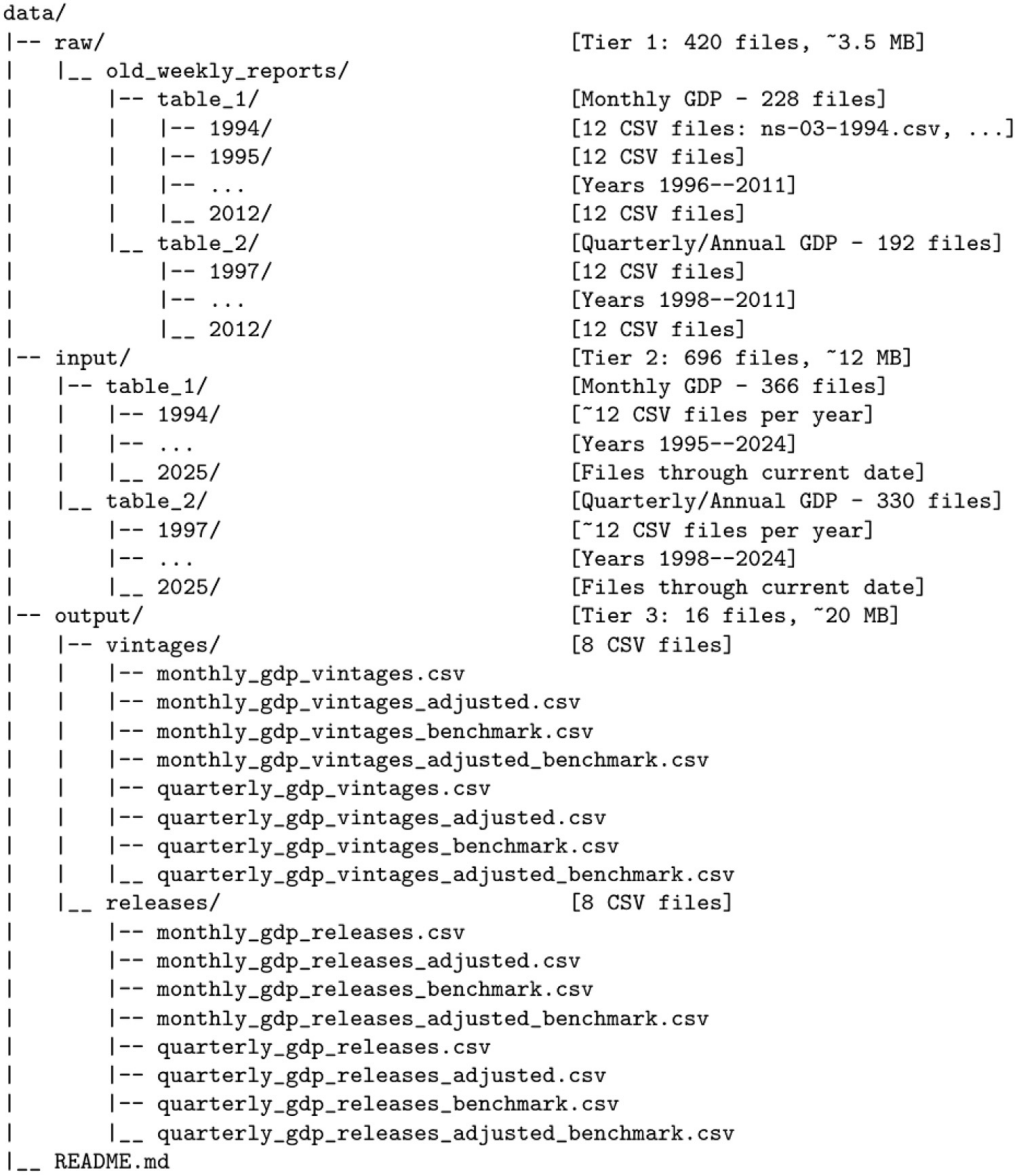
Dataset folder structure and file organization.

Fig. 1 presents the hierarchical organization of the dataset. The three tiers correspond to distinct stages in the data processing pipeline: (1) manually-curated raw data from pre-2013 scanned Weekly Reports, (2) cleaned and standardized input vintages organized by year, and (3) concatenated final output datasets in two complementary formats.

Table 1 summarizes the contents of each data tier.

**Table 1:** Dataset tier summary.

Tier	Folder	Files	Size	Coverage	Description
1	raw/old_weekly_reports/	420	3.5 MB	1994–2012	Manually curated CSV files generated after OCR processing
2	input/	696	12 MB	1994–2025	Cleaned and standardized vintage files used for data construction
3	output/	16	20 MB	1994–2025	Final analysis-ready datasets for econometric and forecasting applications
**Total**		**1,132**	**35.5 MB**		

*Tier 1: Raw Data (raw/old_weekly_reports/)* The raw data tier contains 420 CSV files representing manually-curated data extracted from pre-2013 scanned Weekly Reports. These files are organized into two subdirectories by table type, with year-based subfolders.

*Subdirectory table_1/ (Monthly GDP):* Contains 228 CSV files across 19 year folders (1994–2012), with approximately 12 files per year corresponding to monthly vintages. Each file is named following the convention ns-[week]-[year].csv (e.g., ns-03-2001.csv for Week 3 of 2001).

*Subdirectory table_2/ (Quarterly/Annual GDP):* Contains 192 CSV files across 16 year folders (1997–2012). Coverage begins in 1997 due to the later introduction of quarterly reporting in the Weekly Reports.

*File format:* CSV files use a semicolon (;) as the field delimiter, are encoded in UTF-8 with BOM, and include bilingual headers (Spanish and English sector names).

*Purpose:* These raw CSVs are required for pipeline execution. Users must download this tier to reproduce the complete data processing workflow.

*Tier 2: Input Vintages (input/)* The input tier contains 696 cleaned and standardized CSV files, representing the intermediate stage between raw extraction and final concatenation. Files are organized by table type and year.

*Subdirectory table_1/ (Monthly GDP):* Contains 366 CSV files across 32 year folders (1994–2025). Each year folder contains approximately 12 vintage files, one per month.

*Subdirectory table_2/ (Quarterly/Annual GDP):* Contains 330 CSV files across 29 year folders (1997–2025).

*File format:* CSV files use a semicolon (;) as the field delimiter, follow a wide format with target-period columns, and apply standardized sector names.

*Purpose:* Input vintages enable partial pipeline reproduction (skipping the cleaning step) and provide transparency into the annual vintage construction process.

*Note on Tiers 1–2:* Users loading files programmatically must specify the appropriate sep parameter (e.g., pd.read_csv(..., sep=‘;’)) when importing data from Tiers 1–2.

*Tier 3: Final Output Datasets (output/).* The output tier contains 16 analysis-ready CSV files (they use a comma separator) organized into two subdirectories representing complementary data formats. Table 2 presents the complete file specifications.

**Table 2:** Output dataset file specifications.

Subdirectory	File Name	Rows	Columns	Size (MB)	Format	Description
**Vintage Format (output/vintages/)**						
	monthly_gdp_vintages.csv	2790	406	3.0	Vintage	Monthly GDP, base variant
	monthly_gdp_vintages_adjusted.csv	2790	406	3.0	Vintage	Monthly GDP, base-year adjusted
	monthly_gdp_vintages_benchmark.csv	2790	406	3.0	Vintage	Monthly GDP, benchmark indicators
	monthly_gdp_vintages_adjusted_benchmark.csv	2790	406	3.0	Vintage	Monthly GDP, adjusted + benchmark
	quarterly_gdp_vintages.csv	2511	141	1.3	Vintage	Quarterly/annual GDP, base variant
	quarterly_gdp_vintages_adjusted.csv	2511	141	1.4	Vintage	Quarterly/annual GDP, adjusted
	quarterly_gdp_vintages_benchmark.csv	2511	141	1.3	Vintage	Quarterly/annual GDP, benchmark
	quarterly_gdp_vintages_adjusted_benchmark.csv	2511	141	1.3	Vintage	Quarterly/annual GDP, adjusted + benchmark
**Releases Format (output/releases/)**						
	monthly_gdp_releases.csv	406	343	0.4	Releases	Monthly GDP, base variant
	monthly_gdp_releases_adjusted.csv	406	343	0.5	Releases	Monthly GDP, base-year adjusted
	monthly_gdp_releases_benchmark.csv	406	343	0.4	Releases	Monthly GDP, benchmark indicators
	monthly_gdp_releases_adjusted_benchmark.csv	406	343	0.4	Releases	Monthly GDP, adjusted + benchmark
	quarterly_gdp_releases.csv	141	343	0.4	Releases	Quarterly/annual GDP, base variant
	quarterly_gdp_releases_adjusted.csv	141	343	0.4	Releases	Quarterly/annual GDP, adjusted
	quarterly_gdp_releases_benchmark.csv	141	343	0.4	Releases	Quarterly/annual GDP, benchmark
	quarterly_gdp_releases_adjusted_benchmark.csv	141	343	0.4	Releases	Quarterly/annual GDP, adjusted + benchmark

### 3.2 Data formats

The dataset is organized in two complementary formats that serve different analytical purposes:

• **Vintage format:** Contains all successive records as originally published by BCRP. This constitutes the real-time dataset (RTD) and preserves the exact chronology of publications.
• **Releases format:** Derived from vintages, reorganized by release sequence for revision analysis. This format facilitates the computation of revision statistics and econometric testing.

Both formats contain identical underlying data; only the organizational structure differs. The vintage format ensures transparency and replicability by preserving information as it appeared at each point in time, while the releases format provides the structure for studying revision patterns.

*Operational distinction* A vintage v is the complete set of GDP estimates available from the BCRP publication issued in month v. Reading a single column of the vintage matrix reconstructs the exact information set available to any analyst at time v. A release h is the hth published estimate of a fixed target period t: the first release (h=1) is the flash estimate, the second release (h=2) is the first revision, and so on. Releases are derived from vintages by pivoting the matrix so that each row collects all successive estimates of the same target period. The unit of observation therefore differs: in the vintage format the unit is a (sector, publication month) pair; in the releases format the unit is a (target period, revision-sequence position) pair. The term *vintage* refers to the column dimension (publication date), while the term *release* refers to the position within the revision sequence for a given target period.

*Temporal coverage: two dimensions* The title and abstract report coverage as “1994–2025,” which refers exclusively to the vintage (publication) dimension: the first Weekly Report collected was published in January 1994, and the most recent vintage included was published in 2025. The target-period dimension begins earlier: because a report published in early 1994 includes December 1993 as its most recently measured period, the earliest target period in the monthly dataset is 1993m12. This is not an inconsistency but a structural feature of all real-time datasets: the first vintage always contains target periods predating its own publication date by the length of the publication lag (approximately two months for BCRP). Table 5 reports target-period ranges explicitly by series and frequency.

### 3.3 Vintage format structure

In the vintage format, each row represents a target period (the reference month, quarter, or year being measured). Each column represents a publication vintage, the set of estimates available from BCRP at the end of a given month. Cell values contain GDP growth rates as published in that specific vintage.

Table 3 illustrates this format using the COVID-19 lockdown period (2020–2021), during which monthly GDP growth rates exhibit large negative values that facilitate visual tracking. The table demonstrates how a single target period (e.g., 2020m03) receives multiple estimates across successive vintages. To aid interpretation, first releases are highlighted in blue, second releases in red, third releases in green, and fourth releases in yellow within each target-period row.

**Table 3:** Dataset of monthly vintages (illustrative excerpt).

Notes: This table illustrates the vintage format of a real-time database. Each column corresponds to a specific data vintage, that is, the set of estimates available in the Weekly Report at the end of a given month. Each row corresponds to a target period (e.g., a specific month for which GDP growth is estimated). The publication lag of the first release is two months.

In the 2020m07 column (July 2020 vintage), the value −16.30 for target period 2020m03 (highlighted in green) represents the third estimate of March 2020 GDP growth. In the same column, the value −32.80 for 2020m05 (highlighted in blue) represents the first estimate of May 2020 GDP growth. As such, by reading vertically down a single column, researchers can reconstruct the exact information set available to a policymaker at that specific historical point in time. Conversely, reading horizontally through a row allows for the tracking of the life cycle of a single target period, from its initial estimate to its final value.

### 3.4 Releases format structure

The releases format reorganizes the same underlying data by changing the unit of organization. Rows still correspond to target periods, but columns now group observations by release sequence rather than by publication vintage. The column labeled 1 (First release) collects the first published estimate for each target period, 2 (Second release) collects the second estimate, and so on, up to H (Last release).

Table 4 presents this alternative structure using the same COVID-19 period data. The mapping between formats can be traced by following the colored cells: the blue cells (first releases) from Table 3, which appear in different vintage columns, are collected together in the 1 column of Table 4. Similarly, red cells (second releases) appear under 2, green cells under 3, and yellow cells under 4.

**Table 4:** Dataset of monthly releases (illustrative excerpt).

Notes: This table presents the release format derived directly from the vintage representation in Table 3. The data are not modified; they are only reorganized to group estimates by release order rather than by publication vintage. Each row corresponds to a target period (e.g., a specific month of GDP growth), and each column reports $y_{t}^{h}$, the hth published release of that target-period estimate.

This reorganization makes revisions explicit and computable. The revision from first to second release is $r_{t}^{1,2}=y_{t}^{2}-y_{t}^{1}$; from second to third release, $r_{t}^{2,3}=y_{t}^{3}-y_{t}^{2}$; and so forth. The H column (Last release) contains the most recent available estimate for each target period, after which no further economically meaningful revisions are observed.

### 3.5 Format selection guide

• **Use vintage format for:** Real-time forecasting exercises, nowcasting applications, simulating historical information sets, and evaluating forecast performance with data actually available at the time.
• **Use releases format for:** Revision analysis, testing rationality and efficiency of preliminary estimates, computing revision statistics, and studying convergence patterns across release sequences.

### 3.6 Data variants

Each output file is available in four variants to support different analytical needs:

*Base Variant* Standard GDP growth rates as published by BCRP, suitable for most applications.

*Adjusted Variant (suffix _adjusted).* Applies sentinel value (-999999.0) to growth rates that become incomparable due to base-year methodological changes (1994, 2007, 2019). Marks invalid observations without removing them, preserving complete revision history while flagging structural breaks.

*Benchmark Variant (suffix _benchmark)* Replaces growth rates with binary indicators (1.0 = benchmark revision with simultaneous update of monthly and quarterly/annual tables; 0.0 = routine update). Enables isolation of methodological revisions from standard data updates.

*Combined Variant (suffix _adjusted_benchmark)* Combines both adjustments for analyses requiring both base-year flagging and benchmark identification.

### 3.7 Dataset statistics

Table 5 presents the complete coverage of the real-time dataset across all economic sectors and frequencies.

**Table 5:** Sectoral GDP coverage in the real-time dataset.

Real-time variable (industry)	Frequency	Target period (t)	Revision horizon (h)
Real GDP	Monthly	1993m12–2025m10	1994m02–2025m12
	Quarterly	1996q4–2025q3	1997m02–2025m12
	Annual	1996–2025	1997m02–2025m12
Agriculture and livestock	Monthly	1993m12–2025m10	1994m02–2025m12
	Quarterly	1996q4–2025q3	1997m02–2025m12
	Annual	1996–2025	1997m02–2025m12
Fishing	Monthly	1993m12–2025m10	1994m02–2025m12
	Quarterly	1996q4–2025q3	1997m02–2025m12
	Annual	1996–2025	1997m02–2025m12
Mining and fuel	Monthly	1993m12–2025m10	1994m02–2025m12
	Quarterly	1996q4–2025q3	1997m02–2025m12
	Annual	1996–2025	1997m02–2025m12
Manufacturing	Monthly	1993m12–2025m10	1994m02–2025m12
	Quarterly	1996q4–2025q3	1997m02–2025m12
	Annual	1996–2025	1997m02–2025m12
Electricity and water	Monthly	2003m04–2025m10	2003m06–2025m12
	Quarterly	2003q2–2025q3	2003m08–2025m12
	Annual	2003–2025	2004m02–2025m12
Construction	Monthly	1993m12–2025m10	1994m02–2025m12
	Quarterly	1996q4–2025q3	1997m02–2025m12
	Annual	1996–2025	1997m02–2025m12
Commerce	Monthly	1993m12–2025m10	1994m02–2025m12
	Quarterly	1996q4–2025q3	1997m02–2025m12
	Annual	1996–2025	1997m02–2025m12
Other services	Monthly	1997m05–2025m10	1997m07–2025m12
	Quarterly	1997q2–2025q3	1997m09–2025m12
	Annual	1997–2025	1997m03–2025m12

Notes: Industry disaggregation follows level 9 of the INEI official classification. “Other services” comprises transportation, telecommunications, financial services, real estate, public administration, and related activities.

### 3.8 Data schema

*Vintage Format Schema*

• industry (string): Standardized sector name (9 categories: agriculture, fishing, mining, manufacturing, electricity, construction, commerce, services, gdp).
• vintage (string): Publication month in YYYYmM format (e.g., 2020m5 for May 2020).
• tp_YYYYmM, tp_YYYYqQ, or tp_YYYY (float): GDP growth rate columns for each target period.

*Releases Format Schema*

• target_period (string): Reference period in YYYYmM, YYYYqQ, or YYYY format.
• [sector]_[h] (float): The hth release for each sector (e.g., agriculture_1, agriculture_2,..., gdp_12).

### 3.9 File naming conventions

Files follow consistent naming conventions across all tiers:

• **Raw and input files:** ns-[week]-[year].csv, where ns denotes “Nota Semanal” (Spanish for Weekly Report), week is the report number (01–52), and year is the four-digit year.
• **Output files:** [frequency]_gdp_[format]_[modifiers].csv, where frequency is monthly or quarterly; format is vintages or releases; and optional modifiers are adjusted, benchmark, or adjusted_benchmark.

### 3.10 Technical specifications

• File format: CSV (comma-separated for output, semicolon-separated for raw/input).
• Encoding: UTF-8.
• Missing values: Empty cells or NaN.
• Decimal separator: Period (.).
• Temporal coverage (vintage/publication dimension): 1994–2025 (31 years; monthly vintages from 1994m1 to 2025m10).
• Temporal coverage (target-period dimension): begins from 1993m12 (monthly) and 1993q4 (quarterly), as the first 1994 publications report on December 1993 as the most recently measured period. The title “1994–2025” refers to the vintage dimension.
• Vintages tracked: 310 monthly vintages (1994m1–2025m10).
• Economic sectors: 9 (aggregate GDP plus 8 industries).
• Total observations: 2,561+ sector-period-vintage combinations per frequency.

Sentinel value -999999.0 in adjusted variants marks observations affected by base-year changes, not true missing values.

Variable dictionary summary. Table 6 below provides a concise variable dictionary covering units, missing-value treatment, and flags for all output variables. A complete and navigable version is available at https://github.com/JasonCruz18/peru_gdp_revisions/blob/main/docs/DATA_DICTIONARY.md.

**Table 6:** Variable dictionary for output datasets.

Variable	Type	Unit	Notes
industry	string	–	Standardized sector code (9 values: gdp, agriculture, fishing, mining, manufacturing, electricity, construction, commerce, services). Based on INEI level-9 classification.
vintage	string	–	Publication month in YYYYmM format (vintage format only).
target_period	string	–	Reference period in YYYYmM, YYYYqQ, or YYYY format (releases format only).
tp_YYYYmM / tp_YYYYqQ / tp_YYYY	float	% (y-o-y)	GDP growth rate as year-over-year percentage change; non-annualized; reported to 1 decimal place (e.g., 2.3, −15.3).
[sector]_k	float	% (y-o-y)	kth published release for a given sector and target period (releases format only).
**Special values**			
NaN	float	–	Data not yet published at that vintage, or no further revision exists. NaN≠ zero growth (zero growth is coded 0.0).
-999999.0	float	flag	Sentinel value in _adjusted variants only. Marks observations rendered non-comparable by a base-year methodology change. Not a missing value; must be excluded before analysis.
0.0 / 1.0	float	flag	Benchmark indicator in _benchmark variants: 1.0 = benchmark revision (simultaneous update of monthly and quarterly/annual tables); 0.0 = routine update.

Sector taxonomy over time. Sector definitions follow INEI’s level-9 classification throughout the dataset. Naming conventions changed across vintages (e.g., “Minería” vs. “Minería e Hidrocarburos”); all historical variants are mapped to the standardized English codes listed above via the sector name concordance table in docs/DATA_DICTIONARY.md. No sectors were added or removed across the sample period; differences across vintages reflect labeling changes only, not structural reclassifications.

## 4 Experimental Design, Materials and Methods

### 4.1 Data sources

*Primary Source* Banco Central de Reserva del Perú (BCRP) Weekly Reports (*Nota Semanal*, in Spanish), published every Friday since 1994. Each report reproduces GDP growth statistics from Peru’s National Institute of Statistics and Informatics (Instituto Nacional de Estadística e Informática (INEI)) in standardized tables.

*Historical Context* Prior to 2013, Weekly Reports existed only as hardcover volumes stored at the BCRP’s Renzo Rossini Library (Lima, Peru). From 2013 onwards, reports became freely available as digital PDFs at https://www.bcrp.gob.pe/publicaciones/nota-semanal.html.

### 4.2 Revision calendar

Peru lacks an official GDP revision calendar. We reconstructed an implicit monthly revision calendar by systematically collecting the last Weekly Report of each month, which defines a data vintage. This approach is justified by: (1) INEI Chief Resolution No. 316-2003-INEI mandating quarterly updates, and (2) empirical observation that Weekly Reports update GDP at least monthly.

### 4.3 Data collection methods

Data collection employed two distinct methodologies depending on time period:

#### 4.3.1 Pre-2013 data (Scanned PDFs: OCR-based Extraction)

*Physical Access* Hardcover Weekly Report volumes were consulted in person at the BCRP Renzo Rossini Library. Volumes were photocopied or photographed (no removal permitted), producing approximately 228 scanned PDF files covering 1994–2012.

*OCR Pipeline Implementation* To demonstrate methodology transparency, we developed a comprehensive OCR pipeline and processed year 2001 (12 PDF files) as a complete worked example. This year was selected for its relatively clean scan quality and bilingual format (Spanish/English) representative of post-2000 publications. The complete pipeline, implemented in the OCR/ directory, consists of 11 preprocessing steps:

1. PDF-to-PNG conversion: render pages at 300 DPI using the pdf2image library.
2. Grayscale conversion: transform color images to reduce computational complexity.
3. Adaptive binarization: apply Otsu’s method for text-background separation.
4. Noise removal: median filtering (3×3 kernel) to eliminate speckles and smudges.
5. Skew correction: Hough line transform for rotation detection; critical fix to reject angles near $\pm 90^{\circ }$ (prevents incorrect rotations from table grid lines).
6. DPI scaling: ensure minimum 300 DPI resolution.
7. CLAHE enhancement: Contrast Limited Adaptive Histogram Equalization (clip=2.0, grid=8x8) for faint characters.
8. Morphological thinning: optional character separation (disabled by default).
9. Border removal: crop 10-pixel borders to eliminate edge artifacts.
10. Table extraction: isolate upper table (growth rates) by cropping to the top 50% of page.
11. OCR execution: Tesseract 5.x with Spanish and English models (PSM=6, OEM=3).

*Post-processing Enhancements*

• Bilingual sector recognition: parser identifies sectors in Spanish (*Agropecuario, Pesca*) and English (*Agriculture, Fishing*).
• Fuzzy matching: tolerates OCR character errors (e.g., “agropeciano” → “agropecuario”, “menera” → “minería”) using edit-distance matching with 2-character tolerance.
• Confidence scoring: Tesseract word-level confidence combined with validation checks; outputs below the 85% threshold are flagged for review.
• Quality validation: automated checks for expected sector count, numeric ranges, and CSV structure.

*Year 2001 Demonstration Results*

• Processing success: 100% (12/12 PDFs converted to CSV).
• Data completeness: 70.5% (755 missing / 2561 total cells).
• Sectors extracted: 114 sector-month observations.
• Processing time: approximately 3 min (around 15 s per PDF).
• OCR errors: common character substitutions documented (e.g., “agropacuaro”, “posci”), demonstrating the need for manual review.

Automated OCR outputs stored in OCR/output/table_1/2001/ serve as transparent documentation of initial extraction. However, extensive manual correction was required for production quality.

*Quality Challenges* Scanned sources exhibited warped text, misaligned tables, shadowing, blur, highlighted entries, inconsistent fonts, and residual marks obscuring digits or decimal points. These issues necessitated extensive manual verification.

*Manual Verification and Curation* Each OCR-extracted table was:

1. Compared against the original scanned PDF by human reviewers.
2. Corrected for character recognition errors (e.g., “9.4” read as “5.4”, “O” → “0”, “l” → “1”).
3. Validated for missing values and structural inconsistencies.
4. Standardized for sector names and numeric reasonableness.
5. Stored as the final curated CSV in data/raw/old_weekly_reports/.

These manually-curated CSV files represent the official “raw data” for pre-2013 periods. The year 2001 OCR demonstration, complete pipeline code, and documentation are publicly available in the OCR/ directory, providing full transparency about data collection methodology while acknowledging that human intervention was necessary for quality assurance.

*Formal Correction Criteria* A correction was applied to an OCR-extracted cell whenever: (i) any extracted numeric digit differed from the digit visible in the scanned original; (ii) a sector label was absent, truncated, or misrecognized; or (iii) a structural element, such as row ordering, column alignment, or header, did not match the source document. Isolated illegible entries (e.g., cells fully obscured by physical damage or extreme shadowing) were retained as NaN and flagged in the review log as “source illegible,” distinguishing them from OCR recognition errors.

*Quality Control Protocol* Each vintage file underwent two independent review passes conducted by human reviewers with access to the original scanned PDF. In the first pass, the reviewer compared every extracted cell against the source document and annotated all discrepancies. In the second pass, a second reviewer independently verified that every annotated correction had been applied and that no additional errors remained. A file was promoted to the curated data/raw/old_weekly_reports/ directory only after both passes reached full agreement on all values. In practice, inter-pass disagreement was resolved by direct comparison with the printed source, with the source document serving as the definitive ground truth.

*Traceability* Corrections are traceable at two levels. First, the version-controlled GitHub repository (https://github.com/JasonCruz18/peru_gdp_revisions) preserves the full commit history of the curated CSV files, recording the date and a description of each correction applied. Second, the unmodified OCR outputs for year 2001 are preserved in OCR/output/table_1/2001/ and serve as a transparent baseline against which manual corrections can be directly compared. The OCR/MANUAL_REVIEW_GUIDE.md document describes the review protocol in full detail, including the criteria, reviewer roles, and escalation procedure for ambiguous cases.

*Automated Cleaning.* Curated pre-2013 tables were processed using Python module peru_gdp_rtd.cleaners.old_table_cleaner with orchestration functions old_table_1_runner and old_table_2_runner.

#### 4.3.2 Post-2013 data (Digital PDFs: Automated Pipeline)

*Web Scraping* Automated download of PDF reports using Selenium WebDriver (version 4.0+) with Chrome browser. The scraper implements rate limiting, retry logic with exponential backoff, and concurrent downloads while respecting server policies.

*PDF Table Extraction* Tables were extracted using Tabula-py (version 2.5+), a Python wrapper for the Tabula Java library. Keyword-based page filtering identifies relevant GDP tables before extraction.

*Common Extraction Issues* Digital PDFs presented encoded characters (e.g., (cid:101)), incomplete table borders, duplicate documents, empty cells misinterpreted as headers, missing year labels, and fragmented borders.

*Automated Cleaning* Post-2013 tables were processed using Python module peru_gdp_rtd.cleaners.new_table_cleaner with orchestration functions new_table_1_runner and new_table_2_runner.

### 4.4 Data processing pipeline

The complete pipeline is implemented in Python 3.10+ and organized into 14 specialized modules totaling 4000+ lines of documented code. All processing is configuration-driven via the YAML file config/config.yaml, eliminating hardcoding.

*Pipeline Architecture*

1. **Configuration module** (peru_gdp_rtd.config): type-safe settings management with Pydantic-style validation.
2. **Scrapers module** (peru_gdp_rtd.scrapers): Selenium-based PDF downloading with authentication, rate limiting, and retry logic.
3. **Processors module** (peru_gdp_rtd.processors): PDF processing, keyword extraction, metadata parsing.
4. **Cleaners module** (peru_gdp_rtd.cleaners): 70+ specialized cleaning functions organized in 7 sub-modules:
  • text_cleaners (4 functions): Unicode normalization, whitespace handling.
  • table_cleaners (22 functions): DataFrame operations, noise removal.
  • column_handlers (14 functions): Column renaming, type conversion.
  • table1_cleaners (13 functions): Monthly table transformations.
  • table2_cleaners (13 functions): Quarterly/annual table transformations.
  • old_table_cleaner: orchestrator for pre-2013 data.
  • new_table_cleaner: orchestrator for post-2013 data.
5. **Transformers module** (peru_gdp_rtd.transformers):
  • vintage_preparator: reshapes tables to vintage format.
  • concatenator: merges vintages across years (372 lines).
  • metadata_handler: base-year mapping and benchmark indicators (655 lines).
  • releases_converter: vintage-to-releases transformation (241 lines).
6. **Orchestration module** (peru_gdp_rtd.orchestration): high-level workflow coordination.
7. **Utilities module** (peru_gdp_rtd.utils): RecordManager for idempotent operations and alerts.

*Execution* The complete pipeline is executed via a single command: python scripts/update_rtd.py. It supports selective step execution, dry-run mode, and custom configuration files.

### 4.5 Data cleaning procedures

*Text Normalization*

• Unicode normalization (NFC form).
• Whitespace standardization (tabs → spaces).
• Special character handling.
• Case normalization for sector names.

*Table Structure Cleaning*

• Empty row removal (NA or whitespace-only).
• Duplicate detection and removal.
• Header row extraction.
• Column alignment correction.
• Numeric column identification.

*Numerical Data Cleaning*

• Encoded character replacement.
• Decimal separator normalization (comma → period).
• Thousands separator removal.
• String-to-float conversion with error handling.
• Missing value handling (empty strings, dashes → NaN).

*Temporal Data Handling*

• Missing year label inference via forward-filling.
• Spanish month abbreviation parsing (ene, feb, mar,...).
• Quarter identification (I, II, III, IV).
• Target period construction (e.g., 2020m5, 2020q2).

*Sector Name Standardization* Comprehensive mappings handle Spanish and English terminology variations across vintages (e.g., *agropecuario* → *agriculture, minería e hidrocarburos* → *mining*).

### 4.6 Base-year adjustments

Over the 30-year sample period, Peru’s GDP series underwent three base-year changes (1994, 2007, 2019), creating structural breaks. We identify affected periods via metadata mapping and apply sentinel value (-999999.0) to mark incomparable observations. This preserves complete revision history while flagging invalid comparisons.

### 4.7 Benchmark indicators

Benchmark revisions occur when INEI simultaneously updates monthly and quarterly/annual tables, typically due to methodological changes. We identify these events by comparing release dates across table types and generate binary indicators (1.0 = benchmark, 0.0 = routine update).

### 4.8 Format conversion

Vintage-to-releases conversion is implemented via a NumPy-based vertical alignment algorithm. For each target period, the algorithm extracts all releases chronologically and aligns them in revision-sequence columns.

### 4.9 Quality validation

Automated validation checks include:

• Schema validation (expected columns, correct types).
• Range validation (growth rates within plausible bounds ±50%). Values exceeding this threshold are not automatically discarded; instead, the pipeline flags them as warnings and logs them for human review. The reviewer then inspects the flagged cells against the original source PDF to distinguish genuine extreme observations (e.g., sectoral contractions exceeding −40% during the 2020 COVID-19 lockdown) from extraction errors. Confirmed extreme values are retained as-is; extraction errors are corrected and reprocessed.
• Continuity checks (vintage date monotonicity).
• Completeness checks (missing value patterns).

### 4.10 Software and tools

*Core Dependencies*

• Python 3.10+ (programming language).
• pandas 2.0+ (DataFrame operations).
• numpy 1.24+ (numerical computations).
• selenium 4.0+ (web scraping).
• tabula-py 2.5+ (PDF table extraction).
• tesseract-ocr 5.x (OCR engine for pre-2013 data, with Spanish and English packs).
• pytesseract 0.3+ (Python wrapper for Tesseract).
• opencv-python 4.8+ (image preprocessing).
• scikit-image 0.21+ (advanced image processing algorithms).
• pdf2image 1.16+ (PDF-to-PNG conversion).
• Pillow 10.0+ (image manipulation).
• PyMuPDF 1.23+ (PDF metadata extraction).
• tqdm 4.65+ (progress tracking).
• pyyaml 6.0+ (configuration parsing).

*Development Environment*

• Operating system: Windows 10, macOS, Linux (cross-platform tested).
• Java Runtime Environment 8+ (required for Tabula).
• Git for version control.
• GitHub Actions for continuous integration.

*Hardware Requirements*

• CPU: multi-core processor recommended.
• RAM: 8 GB recommended.
• Disk: 5 GB for full dataset archive.
• Internet: stable connection for PDF downloads.

### 4.11 Data license and reuse conditions

The dataset (all CSV files deposited on Zenodo) is released under the Creative Commons Attribution 4.0 International (CC BY 4.0) license. Copyright © 2025 Jason Josue Cruz, Diego Winkelried, and Javier Torres.

Under CC BY 4.0, any user is permitted to freely copy, redistribute, adapt, remix, and build upon the dataset for any purpose, including commercial use, provided that appropriate credit is given to the original authors (see citation instructions in the Zenodo repository), a link to the license is provided (https://creativecommons.org/licenses/by/4.0/), and any modifications are indicated. No additional restrictions may be imposed on recipients of the material.

The accompanying code repository is released under the MIT License, which similarly permits unrestricted use, reproduction, and modification, subject to inclusion of the original copyright notice. License terms for both the data and the code are explicitly stated in the Zenodo repository and the GitHub repository, respectively.

### 3.12 Minimum reproducible steps

To reproduce the complete dataset from scratch using the frozen code release (GitHub Release v1.0.0; DOI: https://doi.org/10.5281/zenodo.18792757), follow these minimum steps:

1. Obtain the frozen code: clone or download the v1.0.0 release from https://github.com/JasonCruz18/peru_gdp_revisions/releases/tag/v1.0.0.
2. Install dependencies: create a Python 3.10+ environment and run pip install -r requirements-frozen.txt (pinned versions). Java JRE 8+ must also be installed for PDF extraction.
3. Obtain pre-2013 raw data: download the raw/ and input/ directories from the Zenodo repository (https://doi.org/10.5281/zenodo.18099975) and place them under data/raw/old_weekly_reports/ and data/input/, respectively. These files cannot be regenerated automatically because the source scanned PDFs require in-person library access.
4. Configure the pipeline: copy config/config.example.yaml to config/config.yaml. Default values are sufficient for full replication.
5. Run the pipeline: execute python scripts/update_rtd.py from the repository root. The pipeline downloads all post-2013 PDFs automatically, processes them, and regenerates all 16 output datasets in data/output/.
6. Verify outputs: run python scripts/validate_rtd.py to confirm schema compliance and range validation.

Estimated runtime is 45–60 min for a full run from scratch. Users wishing to skip the PDF download step (Steps 1–2 of the pipeline) may instead use the pre-processed Tier 2 input vintages from Zenodo and run only Steps 3–6 (python scripts/update_rtd.py -steps 3,4,5,6), which completes in approximately 5–10 min.

By reusing or redistributing these outputs, users accept the CC BY 4.0 terms described in the Data License section above.

The following infrastructure further supports full reproducibility of the pipeline: MIT License on the code (permits unrestricted academic and commercial use); configuration-driven execution with no hardcoded paths or parameters; six step-by-step tutorial Jupyter notebooks; comprehensive documentation (130+ pages); continuous integration testing across platforms and Python versions (3.10–3.12); and machine-readable citation metadata (CITATION.cff).

## Limitations

This dataset has several limitations stemming from source data characteristics and collection constraints:

*OCR Methodology Demonstration.* While the complete OCR pipeline is publicly available and was successfully demonstrated on year 2001 (12 PDFs achieving 70.5% automated completeness), we provide the OCR code and year 2001 outputs as methodological documentation. The official raw data for all pre-2013 periods are the manually-curated CSV files in data/raw/old_weekly_reports/, which underwent extensive human verification after OCR extraction. Users should note that OCR accuracy varied across years depending on scan quality.

*OCR Accuracy.* Pre-2013 data extraction via OCR is subject to recognition errors despite extensive preprocessing and manual verification. Poor scan quality (warping, shadowing, blur) in some hardcover volumes necessitated more intensive manual correction. All OCR outputs underwent human review before inclusion in the final dataset. OCR completeness and accuracy varied across years depending on scan quality, binding condition, and the bilingual format of the source volumes (post-2000 volumes, available in both Spanish and English, tended to produce higher OCR accuracy than pre-2000 volumes). The year 2001 demonstration (70.5% automated completeness) is representative of the post-2000 period; users should expect higher manual intervention rates for pre-2000 vintages.

• **Automatically replicable:** All 11 preprocessing steps (PDF-to-image conversion, binarization, skew correction, OCR execution) and post-processing enhancements (fuzzy matching, confidence scoring) are fully automated via the code in OCR/. Any user can run python scripts/run_ocr_pipeline.py to reproduce the raw OCR output for year 2001.
• **Human-dependent:** The manual correction step (two-pass review protocol described in the Methods section) requires human judgment and access to the original scanned PDFs. This step cannot be automated. The final curated CSVs in data/raw/old_weekly_reports/ are the authoritative outputs of this human-in-the-loop process.

*Publication Irregularities* While we reconstructed a monthly revision calendar, actual Weekly Report publication was sometimes irregular, particularly in early years. Missing or incomplete reports may create gaps in vintage coverage.

*Sectoral Coverage* Sector definitions and naming conventions changed over time, requiring manual mapping. Some sectors were not reported consistently across all vintages, resulting in missing values for certain sector-period-vintage combinations.

*Base-Year Changes* Three base-year revisions (1994, 2007, 2019) create structural breaks making cross-period comparisons invalid. While we flag affected observations, users must carefully handle these discontinuities in analysis.

*Metadata Completeness* Revision metadata (reasons for revisions, methodological changes) are not systematically documented by the source and therefore not included in this dataset. Benchmark indicators identify simultaneous table updates but cannot distinguish specific methodological changes.

*Representativeness* This dataset reflects only officially published GDP statistics. Unpublished revisions or corrections are not captured.

## Ethics Statement

The authors confirm that this work does not involve human subjects, animal experiments, or data collected from social media platforms. The dataset contains no personally identifiable information (PII) of any kind; all variables are aggregate macroeconomic statistics reported at the national or sectoral level. All data are derived from publicly available official government publications (BCRP Weekly Reports), which reproduce statistics from Peru’s Instituto Nacional de Estadística e Informática (INEI). No ethical approval was required for this data compilation work. The authors have read and followed the ethical requirements for publication in *Data in Brief*.

## CRediT Authorship Contribution Statement

**Jason Josue Cruz:** Conceptualization, Data curation, Formal analysis, Investigation, Methodology, Software, Validation, Visualization, Writing – original draft, Writing – review & editing; **Diego Winkelried:** Conceptualization, Formal analysis, Investigation, Methodology, Validation, Visualization, Writing – original draft, Supervision, Writing – review & editing, Funding acquisition; **Javier Torres:** Conceptualization, Formal analysis, Writing – original draft, Supervision, Writing – review & editing, Funding acquisition.

## Data Availability

ZenodoPeru GDP Real-Time Dataset (1994-2025): Tracking Three Decades of Revisions.

## References

[1] Croushore D. (2011). Frontiers of real-time data analysis. J. Econ. Lit..

[2] Mankiw N.G., Shapiro M.D. (1986). News or noise: an analysis of GNP revisions. Surv. Curr. Bus..

[3] Aruoba S.B. (2008). Data revisions are not well behaved. J. Money Credit Bank..

[4] Strohsal T., Wolf E. (2020). Data revisions to German national accounts: are initial releases good nowcasts?. Int. J. Forecast..

[5] Croushore D., Stark T. (2001). A real-time data set for macroeconomists. J. Econom..

[6] Croushore D., Elliott G., Granger C.W.J., Timmermann A. (2006). Handbook of Economic Forecasting.

[7] Kishor N.K. (2011). Data revisions in India: implications for monetary policy. J. Asian Econ..

[8] Ramos R.L.O., Robitaille P.T., de la Rocque Palis R. (2004). International Finance Discussion Papers.

[9] Clark T.E., McCracken M.W., Elliott G., Timmermann A. (2013). Handbook of Economic Forecasting.

